# Introducing *BMC Global and Public Health*

**DOI:** 10.1186/s44263-023-00012-7

**Published:** 2023-07-31

**Authors:** Gerrit John-Schuster

**Affiliations:** Springer Nature, BioMed Central, New York, NY USA

*BMC Global and Public Health published its first articles today. The journal provides an open-access, transparent peer review forum to promote health and well-being, support equity, diversity and inclusivity in health and inform policy-making worldwide. In doing so, we pledge to serve all global and public health communities.*

BMC, the first open-access (OA) publisher, has always been a champion of open science and data sharing and has long had a strong representation and excellent standing within the public health communities. Indeed, BMC Public Health, a member of the BMC Series, has been a trusted and inclusive outlet for all sound science content within this space for more than two decades. During the COVID-19 pandemic, and other infectious disease outbreaks and related public health emergencies, the scientific community was in high demand for providing robust answers to the most pressing questions and concerns of the public. To advance knowledge and decision-making and to enhance trust in health research and policy, we have launched *BMC Global and Public Health* as a venue for high-quality research advances from all global and public health fields. By connecting all involved research communities, we anticipate the journal to have global reach and impact, improving health and well-being for all.

The journal covers a broad scope and considers work that represents a significant advance and contribution in the field. We are inclusive of all research topics, covering well-established as well as emerging areas in global and public health. We further encourage collaboration across multiple stakeholders, to ensure that we recognize and amplify the voices of historically underrepresented populations in health research and scientific publishing. Addressing health equity issues is at the core of our journal mission. This is illustrated by some of the content we published today, which includes two primary papers: one investigating barriers to cervical cancer screening in Rwanda [1] and another exploring the prevalence of child marriage and its association with partner controlling behaviours in sub-Saharan Africa [2].

We aim to engage our research communities in open discussion about new or controversial findings and opinions by publishing a set of high-impact commissioned content including comment, review and perspective articles. Today, we published two comments, one exploring the role of community healthcare workers in addressing gender-based vulnerabilities during the devastating floods in Pakistan in 2022 and lessons learned for future natural disasters [3]. In the second comment, West et al. highlight communication strategies that can promote vaccination behaviours in sub-Saharan Africa [4].

We have launched the journal with a collaborative editorial model, meaning that we engage our editorial board in the journal’s day-to-day efforts such as manuscript submissions, collection topics and other relevant journal decisions and activities, including community outreach and soliciting content. To achieve this, we are actively recruiting editorial board members (EBMs) to strengthen our connections with the research communities and to support the field. In doing so, we always strive for diversity in backgrounds and in the representation of geographical regions. We are pleased that our efforts have brought together an engaged group of people from all over the world, covering topic areas such as infectious and noncommunicable disease epidemiology, maternal and child health, environmental and occupational health, genetic screening, disease modeling and more. In these two Q&As, four of our EBMs, Angeline Ferdinand, Stephanie Lo, Michael Murray and Robert Paulino-Ramírez, have taken the time to share insights into their research careers and areas of expertise, the main problems that need addressing in their fields, and how their role at the journal can help advance these areas [5, 6]. We are excited about expanding our editorial board as the journal grows.

To spotlight high priority research areas and to explore emerging ones, we collaborate with guest editors on article collections that cover topics of interest to the journal and its research communities. We encourage our EBMs and other researchers to suggest and lead collections in their fields of research, and we provide the support and the means to reach as many researchers as possible so that we can showcase the important advances made in global and public health. When we opened for submissions, we launched two article collections that cover highly topical issues: *Stigma and mental health in infectious diseases*, guest-edited by Amrita Daftary and Jeremiah Chikovore, and *Identifying people with tuberculosis and linking to care: finding the missing millions*, guest-edited by Rachael Burke and Finn McQuaid. In this Q&A, Rachael Burke and Finn McQuaid elaborate on the importance of increasing efforts to identify people with tuberculosis to meet End TB goals and how the collection can provide a forum for discussion of recent advances [7]. We look forward to adding the first articles to these collections within the next months. Please also look out for new call for papers at the journal, including for this recently launched collection on *Addressing public health concerns in incarceration and community corrections*, guest-edited by Charlie Brooker.

We are grateful for all the trust we have received from the communities so far, including from all our EBMs, authors and reviewers. And we could not be more proud of the first content that we published today and of becoming a respected global and public health journal for all. We look forward to what the future holds for the journal and its communities.

## Data Availability

Not applicable.
